# C–N exchange model of legume–*Rhizobium* symbiosis incorporating ATP budget constraints and energy–mass balance between the species

**DOI:** 10.1371/journal.pone.0349611

**Published:** 2026-05-22

**Authors:** Toshiko Furukawa, Takuya Iimura

**Affiliations:** 1 Department of Biological Sciences, Tokyo Metropolitan University, Hachioji, Tokyo, Japan; 2 Department of Economics and Business Administration, Tokyo Metropolitan University, Hachioji, Tokyo, Japan; Nepal Agricultural Research Council, NEPAL

## Abstract

We present a comparative advantage model of carbon–nitrogen exchange in legume–*Rhizobium* symbiosis that incorporates ATP budget constraints and the energy–mass balance between the host and symbiont. In this framework, the uptake of carbon and nitrogen is limited by the ATP available to each partner, and any imbalance in trade is compensated by adjustments in symbiont biomass. Using empirical estimates of the ATP costs of carbon and nitrogen uptake, together with data on body C:N ratios, the model generates three key predictions, and we prove that they align with empirical results. (i) The condition for the establishment of symbiosis derived from the model is consistent with measured ATP costs in both host and symbiont. (ii) At equilibrium, the model predicts a relatively low carbon supply from the legume and a relatively high nitrogen supply from *Rhizobium*, in agreement with reported patterns of exchange. (iii) The model further predicts that the proportion of carbon supplied decreases as the host C:N ratio increases, and that the proportion of nitrogen supplied decreases as the symbiont C:N ratio decreases, which are consistent with the empirically observed decline in nodulation during host aging.

## 1. Introduction

Carbon (C) and nitrogen (N) are two essential elements for all living organisms, and a shortage of either has profound consequences for growth. For plants, soil N is typically limiting, whereas C is readily available through photosynthesis. The agricultural value of legumes as green manure was already recognized in ancient Rome [1]. In the nineteenth century, agronomists discovered that legumes use atmospheric N as a nutrient source, and that root nodules, which contain “bacteria-like” structures [2], are responsible for this fixation. These structures are now known as symbiotic bacteria of the genus *Rhizobium* and related taxa. Since then, the mechanisms of nodule formation and N fixation have been extensively investigated by plant and microbial biologists, and the legume–*Rhizobium* symbiosis has also attracted increasing attention from researchers in theoretical fields.

The symbiosis proceeds as follows. When soil N is scarce, legumes release flavonoids that trigger *Rhizobium* to produce nod factors. In response, root cells initiate nodule formation and encapsulate the bacteria, which differentiate into bacteroids capable of N fixation. Once nodulation is completed, a mutualistic exchange of C and N is established [3]. This process is driven exclusively by the plants [4]. Extensive biochemical research has clarified the energy–mass balance within each species, including the ATP costs of CO₂ fixation and N₂ reduction [5]. However, how the energy–mass balance is maintained between the partners remains poorly understood.

One promising framework for analyzing such plant–microbe interactions is the microbial market model [6], a class of biological market models [7,8] that applies concepts from economics to explain interspecific cooperation. An early example is the work of Schwartz and Hoeksema [9,10], who introduced the classical theory of comparative (or relative) advantage in economics [11] to the study of plant–microbe symbiosis. This theory of economics claims that if each of two countries (trade partners, e.g., England and Portugal) specializes in the production of a good for which it has a relative advantage (e.g., wool cloth for England and wine for Portugal, to borrow the original example), then trade becomes mutually beneficial. Schwartz and Hoeksema [9] used this theory to show how specialization and trade in C and phosphorus can benefit both plants and mycorrhizal fungi. While their model was purely theoretical and alternative approaches have since been proposed [12–14], the comparative advantage microbial market model of Schwartz and Hoeksema [9] remains a widely cited foundation for theoretical work on plant–microbe symbiosis.

In this study, we extend this line of research by developing a quantitative comparative advantage model of C–N exchange in legume–*Rhizobium* symbiosis. Specifically, we incorporate hypothetical ATP budget constraints and the energy–mass balance between the species, and we derive unit-free equilibrium ratios that can be compared directly with empirical data. For instance, we obtain supply ratios of C by the legume and N by *Rhizobium* as functions of body C:N ratios and the C to N exchange ratio, the latter determined by ATP costs of C and N uptake. This framework enables us to generate empirically testable hypotheses and evaluate them against data accumulated over several decades.

The analysis yields three main implications. (i) The condition for the emergence of symbiosis: consistent with the observation that legume–*Rhizobium* symbiosis occurs only when soil N is limiting [15,16], we show that the relative ATP costs of C and N uptake determine when symbiosis is favored. (ii) The supply ratios of C and N: the model predicts that, at equilibrium, the legume supplies a small fraction of its fixed C, while *Rhizobium* supplies a large fraction of its fixed N. These predictions are consistent with empirical reports of low C supply by legumes and high N supply by rhizobia. (iii) Dependence on body stoichiometry: the model predicts that the C supply ratio decreases as the legume body C:N ratio increases, and that the N supply ratio decreases as the rhizobial body C:N ratio decreases. These results are consistent with the empirically observed decline in nodulation with host aging, and may also explain why legumes, with their characteristically lower body C:N ratios relative to other plants, are predisposed to form such symbioses.

Two distinctive features of our model should be noted. First, unlike the framework of Schwartz and Hoeksema [9], we account for the shift in rhizobial N acquisition from direct uptake to fixation once symbiosis is established. Second, rather than assuming equilibrium is reached by “price” adjustment of the C to N exchange ratio, we assume adjustment occurs through symbiont biomass, which is more appropriate when resources are demanded in fixed proportions. With ATP budget constraints, equilibrium is attained when energy and mass are simultaneously balanced between host and symbiont. The Edgeworth box, a classical economic tool, is adapted here to illustrate this equilibrium.

## 2. The model

Let $\overset{\text{N}}{\underset{L}{x}}\geq \text{0}$ and $\overset{\text{C}}{\underset{L}{x}}\geq \text{0}$ be the amounts of N and C taken up by a unit mass of legume per unit time. We consider the legume per unit mass. We assume that Darwinian fitness $f_{L}$ of legume is a function of $\overset{\text{N}}{\underset{L}{x}}$ and $\overset{\text{C}}{\underset{L}{x}}$ such that


$$\begin{array}{c} f_{L}=\text{min}\left\{\overset{\text{N}}{\underset{L}{k}}\overset{\text{N}}{\underset{L}{x}},\overset{\text{C}}{\underset{L}{\;k}}\overset{\text{C}}{\underset{L}{x}}\right\}, \end{array}$$ (1)

where $\overset{\text{N}}{\underset{L}{k}}>\text{0}$ and $\overset{\text{C}}{\underset{L}{k}}>\text{0}$ are constants. We have $f_{L}=\overset{\text{N}}{\underset{L}{k}}\overset{\text{N}}{\underset{L}{x}}$ if $\overset{\text{N}}{\underset{L}{k}}\overset{\text{N}}{\underset{L}{x}}\leq \overset{\text{C}}{\underset{L}{\;k}}\overset{\text{C}}{\underset{L}{x}}$, and $f_{L}=\overset{\text{C}}{\underset{L}{k}}\overset{\text{C}}{\underset{L}{x}}$ if $\overset{\text{N}}{\underset{L}{k}}\overset{\text{N}}{\underset{L}{x}}\geq \overset{\text{C}}{\underset{L}{\;k}}\overset{\text{C}}{\underset{L}{x}}$: The functional form of Eq 1 represents Liebig’s law of the minimum [17,18]. The constants $\overset{\text{N}}{\underset{L}{k}}$ and $\overset{\text{C}}{\underset{L}{k}}$ can be seen as representing “organismal stoichiometry” [19], assuming the relationships to body C:N ratio as $\text{C}:\text{N}=\frac{\text{1}}{\overset{\text{C}}{\underset{L}{k}}}:\frac{\text{1}}{\overset{\text{N}}{\underset{L}{k}}}$. (Note that efficient uptake of $\overset{\text{N}}{\underset{L}{x}}$ and $\overset{\text{C}}{\underset{L}{x}}$ is such that $\overset{\text{N}}{\underset{L}{k}}\overset{\text{N}}{\underset{L}{x}}=\overset{\text{C}}{\underset{L}{k}}\overset{\text{C}}{\underset{L}{x}}$, namely, $\overset{\text{C}}{\underset{L}{x}}:\overset{\text{N}}{\underset{L}{x}}=\frac{\text{1}}{\overset{\text{C}}{\underset{L}{k}}}:\frac{\text{1}}{\overset{\text{N}}{\underset{L}{k}}}$.).

**Fig 1:**
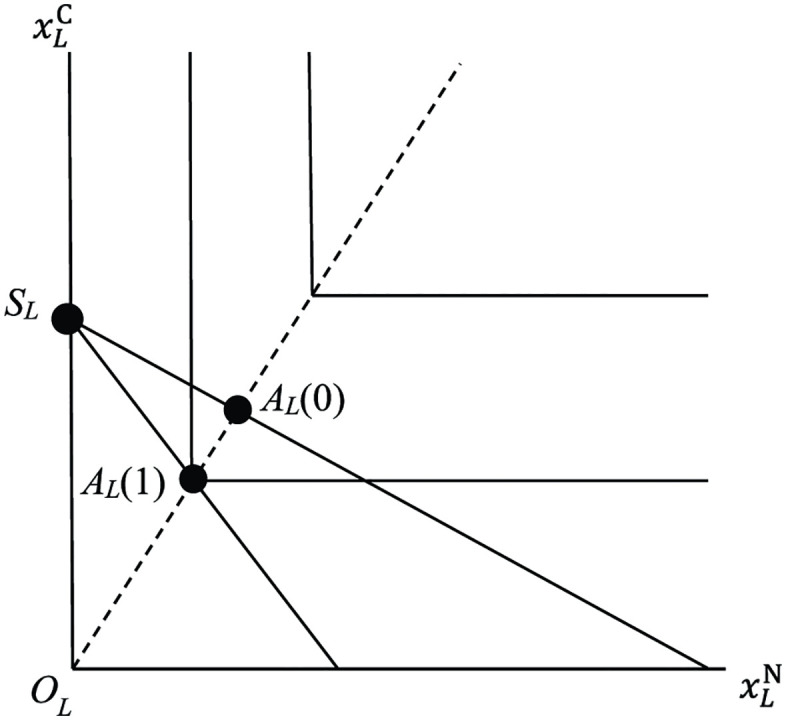
*Autarky points* of legume *A*_*L*_(0) and *A*_*L*_(1). Horizontal axis is uptake of N and vertical axis is uptake of C by a unit mass of legume. The L-shaped lines are contour lines of fitness function and the solid line through the point *A*_*L*_(0) (resp. *A*_*L*_(1)) is *budget line* under soil environment *s* = 0 (resp. *s* = 1) with slope $-r_{L}(\text{0})$ (resp. $-r_{L}(\text{1})$). The dotted line emanating from the origin *O*_*L*_ is *optimality line*. The point *S*_*L*_ is the specialization point of legume.

Legume takes up C by fixing CO_2_ in the air. We assume that unit ATP cost of fixing C is constant and denote it by $\overset{\text{C}}{\underset{L}{a}}>\text{0}$. In a non-symbiotic state, legume takes up N directly from the soil. We assume that unit ATP cost of direct uptake of N depends on the soil environment and denote it by $\overset{\text{N}}{\underset{L}{a}}(s)>\text{0}$, where s∈{0,1} is the soil environment parameter such that s=1 if direct uptake is ATP-demanding, and s=0 if not demanding. Thus, $\overset{\text{N}}{\underset{L}{a}}(\text{1})>\overset{\text{N}}{\underset{L}{a}}(\text{0})$. Assuming that $b_{L}>\text{0}$ is the ATP budget of legume per unit time, uptakes $\overset{\text{N}}{\underset{L}{x}}$ and $\overset{\text{C}}{\underset{L}{x}}$ of non-symbiotic legume must satisfy


$$\begin{array}{c} \overset{\text{N}}{\underset{L}{a}}(s)\overset{\text{N}}{\underset{L}{x}}+\overset{\text{C}}{\underset{L}{a}}\overset{\text{C}}{\underset{L}{x}}=b_{L}, \end{array}$$ (2)

which is the budget constraint of non-symbiotic legume under soil environment *s*. Note that the ratio $r_{L}(s):=\frac{\overset{\text{N}}{\underset{L}{a}}(s)}{\overset{\text{C}}{\underset{L}{a}}}$ represents the cost of N uptake in units of C for legume under soil environment *s*. We assume that non-symbiotic legume chooses $\left(\overset{\text{N}}{\underset{L}{x}},\overset{\text{C}}{\underset{L}{x}}\right)$ in the budget constraint that maximizes its fitness Eq 1. See Fig 1, in which budget constraints under s=0 and s=1 are depicted. We call them *budget lines* (“isolation acquisition isocline” [9]). The absolute values of the slope of budget lines are $r_{L}(\text{0})$ and $r_{L}(\text{1})$, respectively. By definition, $r_{L}(\text{1})>r_{L}(\text{0})$. Non-symbiotic legume chooses point *A*_*L*_(0) if s=0 and *A*_*L*_(1) if s=1, which we call *autarky points* of legume. The dotted line emanating from the origin $O_{L}$ is referred to as *optimality line* of legume (“optimal consumption vector” [9]). The point $S_{L}$ is the *specialization point* of legume, which we will explain later.

Let $\overset{\text{N}}{\underset{R}{x}}\geq \text{0}$ and $\overset{\text{C}}{\underset{R}{x}}\geq \text{0}$ be the amounts of N and C taken up by a population of *Rhizobium* per unit time. We consider a size-variable population of *Rhizobium*. Similarly to legume, we assume that fitness $f_{R}$ of *Rhizobium* is expressed, with organismal stoichiometric constants $\overset{\text{N}}{\underset{R}{k}}>\text{0}$ and $\overset{\text{C}}{\underset{R}{k}}>\text{0}$, by a function


$$\begin{array}{c} f_{R}=\text{min }\left\{\overset{\text{N}}{\underset{R}{k}}\overset{\text{N}}{\underset{R}{x}},\overset{\text{C}}{\underset{R}{\;k}}\overset{\text{C}}{\underset{R}{x}}\right\}. \end{array}$$ (3)

**Fig 2:**
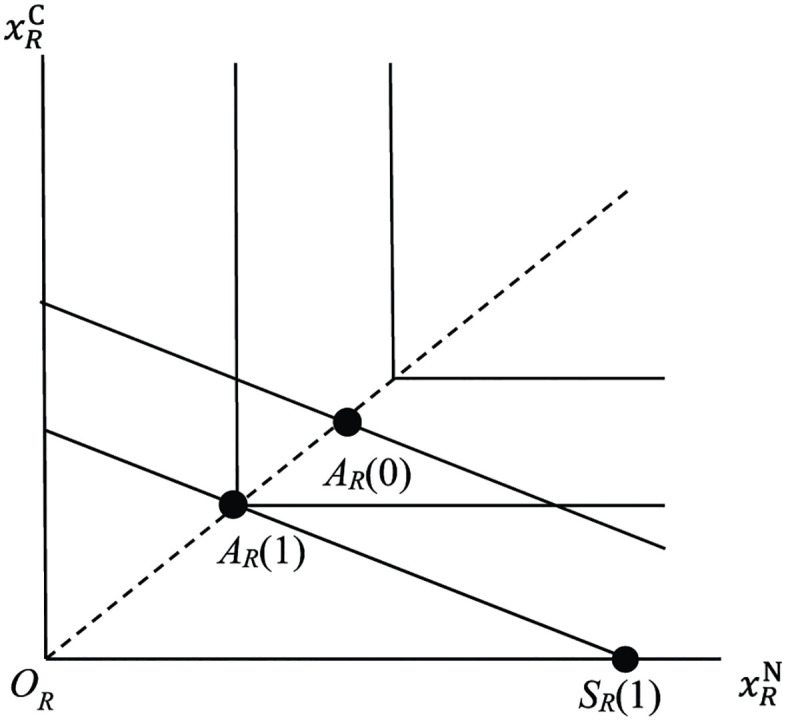
*Autarky points* of *Rhizobium A*_*R*_(0) and *A*_*R*_(1). Horizontal axis is uptake of N and vertical axis is uptake of C by a population of *Rhizobium*. The L-shaped lines are contour lines of fitness function and the solid line through the point *A*_*R*_(0) (resp. *A*_*R*_(1)) is *budget line* under soil environment *s* = 0 (resp. *s* = 1) with slope $-r_{R}$. The dotted line emanating from the origin *O*_*R*_ is *optimality line*. The point *S*_*R*_(1) represents the maximum amount of N that a given population of *Rhizobium* can take by direct uptake when s=1.

In a non-symbiotic state, *Rhizobium* takes up N and C both directly from the soil. We assume that unit ATP costs of direct uptake of N and C both depend on the soil environment s∈{0,1}, and denote them by $\overset{\text{N}}{\underset{R}{a}}(s)>\text{0}$ and $\overset{\text{C}}{\underset{R}{a}}(s)>\text{0}$, with $\overset{\text{N}}{\underset{R}{a}}(\text{1})>\overset{\text{N}}{\underset{R}{a}}(\text{0})$ and $\overset{\text{C}}{\underset{R}{a}}(\text{1})>\overset{\text{C}}{\underset{R}{a}}(\text{0})$. However, since non-symbiotic *Rhizobium* lives in a rhizosphere, a very close neighborhood of legume roots, in which soil C:N ratio is relatively kept constant [20], we assume that the uptake cost of N in units of C for *Rhizobium* is constant (i.e., independent of *s*), and denote it by $r_{R}:=\frac{\overset{\text{N}}{\underset{R}{a}}(s)}{\overset{\text{C}}{\underset{R}{a}}(s)}$. Let $b_{R}>\text{0}$ be the ATP budget of a given population of *Rhizobium* per unit time. We assume that $b_{R}$ is proportionate to the population size, but omit size parameter in the current static analysis for the sake of brevity. The uptakes $\overset{\text{N}}{\underset{R}{x}}$ and $\overset{\text{C}}{\underset{R}{x}}$ of non-symbiotic *Rhizobium* must satisfy


$$\begin{array}{c} \overset{\text{N}}{\underset{R}{a}}(s)\overset{\text{N}}{\underset{R}{x}}+\overset{\text{C}}{\underset{R}{a}}(s)\overset{\text{C}}{\underset{R}{x}}=b_{R}, \end{array}$$ (4)

the budget constraint of non-symbiotic *Rhizobium* under soil environment *s*. We assume that non-symbiotic *Rhizobium* chooses $\left(\overset{\text{N}}{\underset{R}{x}},\overset{\text{C}}{\underset{R}{x}}\right)$ in the budget constraint that maximizes its fitness Eq 3. See Fig 2, in which budget constraints under s=0 and s=1 are depicted. We call them *budget lines*. Due to the assumed constancy of $r_{R}$, budget lines are parallel to each other. Non-symbiotic *Rhizobium* chooses point *A*_*R*_(0) if s=0 and *A*_*R*_(1) if s=1, which are the *autarky points* of *Rhizobium*. The dotted line emanating from the origin $O_{R}$ is *optimality line* of *Rhizobium.* The point $S_{R}(\text{1})$ represents the maximum amount of N that a given population size of *Rhizobium* can take by direct uptake when s=1.

### (a) Trade in symbiosis

**Fig 3:**
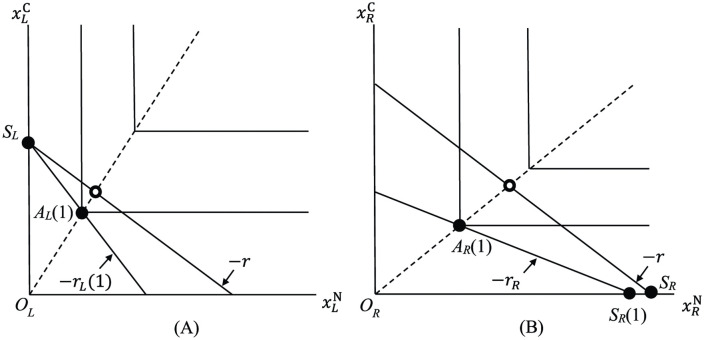
The situation where symbiosis is mutually beneficial. *S*_*L*_ and *S*_*R*_ are specialization points of legume and *Rhizobium*. The lines with slope −r are trade lines. If $r_{L}(\text{1})>r_{R}$ and $\overset{\text{N}}{\underset{R}{b_{R}/a}}(\text{1})\leq b_{R}/\overset{\text{N}}{\underset{R}{\hat{a}}}$, then with exchange ratio r such that $r_{L}(\text{1})>r>r_{R}$, specialization and trade is beneficial for both legume and *Rhizobium*.


The theory of comparative advantage focuses on the ratios $r_{L}(s)$ and $r_{R}$, namely, the costs of N uptake in units of C for legume and *Rhizobium*, and if


$$\begin{array}{c} r_{L}(s)>r_{R}, \end{array}$$ (5)

then it judges that *Rhizobium* has a comparative advantage in the uptake of N (and legume has the one in the uptake of C), and predicts that specialization and trade render benefit for the both [9]. It is our hypothesis that


$$\begin{array}{c} r_{L}(\text{1})>r_{R}\text{ and }r_{R}>r_{L}(\text{0}), \end{array}$$ (6)

namely, the symbiosis is mutually beneficial when s=1, but not when s=0. However, there is a point to be considered in legume–*Rhizobium* symbiosis. As we noted before, *Rhizobium* changes its mode of N uptake from direct uptake to fixation when entering into symbiosis. Thus, although the budget line emanating from *S*_*R*_(1) in Fig 2 is meaningful as the frontier of N-C uptake for non-symbiotic *Rhizobium* when s=1, the point *S*_*R*_(1) cannot be a specialization point of symbiotic *Rhizobium*. In order for the symbiosis to be really beneficial for *Rhizobium*, we need one more condition. Assume s=1 and let $\overset{\text{N}}{\underset{R}{\hat{a}}}>\text{0}$ (“hat” $\overset{\text{N}}{\underset{R}{a}}$) be the unit ATP cost of N fixation by *Rhizobium*, which we assume is constant. Now, if


$$\begin{array}{c} \frac{b_{R}}{\overset{\text{N}}{\underset{R}{a}}(\text{1})}\leq \frac{b_{R}}{\overset{\text{N}}{\underset{R}{\hat{a}}}}, \end{array}$$ (7)

namely, if the amount of N obtained by fixation (the right-hand side) is not less than the amount of N obtained by direct uptake when s=1 (the left-hand side), then, with $r_{L}(\text{1})>r_{R}$ (Eq 5 with s=1), the mutual benefit of specialization and trade is guaranteed. See Fig 3. The budget line of legume is depicted in Fig 3A and that of *Rhizobium* in Fig 3B. The point *S*_*L*_ is *specialization point* of legume and *S*_*R*_ is that of symbiotic *Rhizobium*. In Fig 3B, the coordinate of *S*_*R*_(1) is $\frac{b_{R}}{\overset{\text{N}}{\underset{R}{a}}(\text{1})}$ and the coordinate of *S*_*R*_ is $\frac{b_{R}}{\overset{\text{N}}{\underset{R}{\hat{a}}}}$, so Eq 7 is satisfied. Assuming $r_{L}(\text{1})>r_{R}$, let *r* be an *exchange ratio of C to N* such that

$$\begin{array}{c} r_{L}(\text{1})>r>r_{R}. \end{array}$$ (8)

In our model, this ratio is an exogenously given constant. (See Discussion (b) below regarding the physiological aspect of this ratio). In the figures, the lines with slope −*r* emanating from *S*_*L*_ and *S*_*R*_ are *trade lines* (“trade acquisition isocline” [9]). Under the assumption that legume and *Rhizobium* maximize their own fitness, they choose the intersection point of the trade line and their own optimality lines. Thus, as can be seen from Fig 3, both legume and *Rhizobium* can enjoy uptakes that are superior to their autarky points *A*_*L*_(1) and *A*_*R*_(1), and specialization and trade are beneficial for the both.

**Fig 4:**
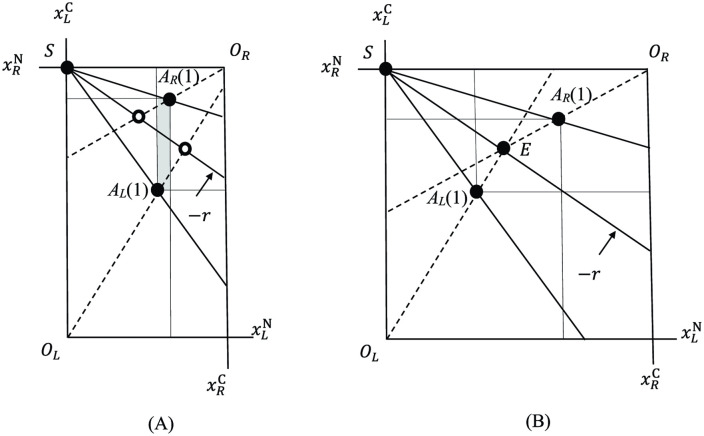
Edgeworth box representation of (A) disequilibrium and (B) equilibrium of trade. The line with slope −r is the trade line. Point *E* in (B) represents the equilibrium of trade, where both legume and *Rhizobium* maximize their fitness on the trade line, and total demand for N equals total supply of N (similarly for C). Note that, at *E*, both legume and *Rhizobium* are strictly better off compared to at their autarky points AL(1) and AR(1). (Here, $\overset{\text{N}}{\underset{R}{b_{R}/a}}(\text{1})=b_{R}/\overset{\text{N}}{\underset{R}{\hat{a}}}$ is assumed).

Fig 4A shows a state in which legume and *Rhizobium* specialize and trade. This is an *Edgeworth’s box diagram* [21], in which Fig 3A and Fig 3B are merged in such a way that the origin *O*_*R*_ of *Rhizobium* is at the top-right corner and the specialization points *S*_*L*_ and *S*_*R*_ of legume and *Rhizobium* are superimposed at the top-left corner, *S*. The height of the box then equals the amount of C fixed by a unit mass of legume. The width equals the amount of N fixed by a given population size of *Rhizobium*. Note that every point in the diagram represents an allocation of fixed N and C to legume and *Rhizobium*, and that any point in the interior of the shaded rectangle in Fig 4A is a mutually beneficial allocation of N and C (compared to staying at their autarky points). In Fig 4A, where demands for N and C by legume and *Rhizobium* are marked by the open circles, there is an excess demand for N (and an excess supply for C), because total N demanded exceeds the width of the box (and total C demanded is less than the height). Thus, some adjustment is needed. We assume that supply–demand imbalance is adjusted by a body mass adjustment, specifically by the adjustment of the population size of *Rhizobium* such that it is increased (resp. decreased) whenever the excess supply (resp. excess demand) of C exists (see Discussion (c) below for the dynamical model of this process). Fig 4B depicts the equilibrium state of such a mass adjustment process. In our current static setting, it is characterized by the intersection of three rays (two optimality lines and the trade line) at some point *E*. There, we have (1) legume and *Rhizobium* both specialize in their products, and, at point *E*, (2) both are maximizing their fitness on the trade line, and (3) supply–demand imbalance is cleared. Thus, point *E* represents a feasible and mutually optimal allocation of N and C. The fitness of both legume and *Rhizobium* are improved at *E* than at their autarky points *A*_*L*_(1) and *A*_*R*_(1). We here refer to *E* as the *equilibrium* of symbiosis.

### (b) Equilibrium values (formulae)

Let *h* be the height of the box of Fig 4B, which is the amount of C fixed by a unit mass of legume; i.e., $h=\frac{b_{L}}{\overset{\text{C}}{\underset{L}{a}}}$. Note that this is equal to the value of $\overset{\text{C}}{\underset{L}{x}}$ at point *S* and constant in the model. Let $c_{L}$ and $c_{R}$ be the slopes of the optimality line of legume and that of *Rhizobium*; i.e., $c_{L}=\frac{\overset{\text{N}}{\underset{L}{k}}}{\overset{\text{C}}{\underset{L}{k}}}$ and $c_{R}=\frac{\overset{\text{N}}{\underset{R}{k}}}{\overset{\text{C}}{\underset{R}{k}}}$. Under our assumption, these are equal to the body C:N ratios of legume and *Rhizobium*, respectively. Using these variables and the exchange ratio *r* in the range of Eq 8, the equilibrium uptake of the N and C by legume, $\left(\overset{\text{N}*}{\underset{L}{x}},\;\overset{\text{C}*}{\underset{L}{x}}\right)$, and that of *Rhizobium*, $\left(\overset{\text{N}*}{\underset{R}{x}},\;\overset{\text{C}*}{\underset{R}{x}}\right)$, are:


$$\begin{array}{c} \left(\overset{\text{N}*}{\underset{L}{x}},\;\overset{\text{C}*}{\underset{L}{x}}\right)=\left(\frac{h}{c_{L}+r},\frac{c_{L}h}{c_{L}+r}\right),\;\left(\overset{\text{N}*}{\underset{R}{x}},\;\overset{\text{C}*}{\underset{R}{x}}\right)=\left(\frac{rh}{c_{R}\left(c_{L}+r\right)},\frac{rh}{c_{L}+r}\right). \end{array}$$ (9)

The equilibrium supplies of C by legume, $\overset{\text{C}*}{\underset{L}{y}}$, and N by *Rhizobium*, $\overset{\text{N}*}{\underset{R}{y}}$, are:


$$\begin{array}{c} \overset{\text{C}*}{\underset{L}{y}}=\overset{\text{C}*}{\underset{R}{x}}=\frac{rh}{c_{L}+r},\;\overset{\text{N}*}{\underset{R}{y}}=\overset{\text{N}*}{\underset{L}{x}}=\frac{h}{c_{L}+r}. \end{array}$$ (10)

The equilibrium width $w^{*}$ of the box, namely, the width of the box of Fig 4B, is:


$$\begin{array}{c} w^{*}=\overset{\text{N}*}{\underset{R}{y}}+\overset{\text{N}*}{\underset{R}{x}}=\frac{\left(c_{R}+r\right)h}{c_{R}\left(c_{L}+r\right)}. \end{array}$$ (11)

See S1 Appendix for the derivations of these formulae.

## 3. Results

### (a) The condition for symbiosis

We now assess the inequalities in Eqs 6–7. Recall that $r_{L}(s)=\frac{\overset{\text{N}}{\underset{L}{a}}(s)}{\overset{\text{C}}{\underset{L}{a}}}$ and $r_{R}=\frac{\overset{\text{N}}{\underset{R}{a}}(s)}{\overset{\text{C}}{\underset{R}{a}}(s)}$. To evaluate each component, we count the number of ATP and ATP equivalents that are required to take up (and convert to if necessary) a specific form of C or N: glyceraldehyde 3-phosphate (G3P) (C_3_H_7_O_6_P) for legume’s C, glucose (C_6_H_12_O_6_) for non-symbiotic *Rhizobium*’s C, and ammonium (NH_4_^+^) for all N. As to the ATP equivalents of reductants, we assume 3 for NADPH, 2.5 for NADH, 1.5 for reduced form of ferredoxin (Fd_red_), by default.

First, during photosynthesis, legume fixes aerial CO_2_ to G3P in Calvin–Benson cycle, in which 9 ATP and 6 NADPH are used for each molecule of G3P [22]. For there are three C in a G3P, ATP cost of C for legume is (9+18)÷3=9 mol ATP/mol C, namely, $\overset{\text{C}}{\underset{L}{a}}=\text{9}$. Non-symbiotic legume’s major N sources are nitrate (NO_3_^-^) and NH_4_^+^ [23]. In aerobic soil, where *Rhizobium* inhabits [24], the dominant form of N is NO_3_^-^ [25]. Since the use of NO_3_^-^ is more ATP-demanding than that of NH_4_^+^, we let $\overset{\text{N}}{\underset{L}{a}}(\text{1})$ and $\overset{\text{N}}{\underset{L}{a}}(\text{0})$ be the ATP cost of uptake of N via NO_3_^-^ and via NH_4_^+^, respectively. Now, NO_3_^-^ is taken up actively by nitrate transporters. Root respiration studies revealed that 1–3 mol ATP are consumed for each molecule of NO_3_^-^ [26]. It is then converted to nitrite (NO_2_^-^) and then to NH_4_^+^ using 1 NAD(P)H and 6 Fd_red_ [22], where 11.5–12 mol ATP/mol N are used. The use of NO_3_^-^ also invokes synthesis of transporter [27] and passing of NO_2_^-^ across plastid membrane that requires energy. Although there is no precise measurement of ATP requirements for them, we may add extra 1–2 mol ATP/mol N, in particular when N is extremely scarce. We thus let $\text{13}.\text{5}\leq \overset{\text{N}}{\underset{L}{a}}(\text{1})\leq \text{17}$. Meanwhile, NH_4_^+^ is taken up passively when its external concentration is high, and actively by using ammonium transporters when the concentration is low [28]. In the former case, the ATP requirement is negligible, but in the latter it requires 1 mol ATP/mol N to use proton pump, so we estimate that 0.5 mol ATP/mol N is needed, i.e., $\overset{\text{N}}{\underset{L}{a}}(\text{0})=\text{0}.\text{5}$. Therefore, with $\overset{\text{C}}{\underset{L}{a}}=\text{9}$, we have $\text{1}.\text{5}\leq r_{L}(\text{1})\leq \text{1}.\text{8}\text{8}$ and $r_{L}(\text{0})=\text{0}.\text{0}\text{5}$.

Second, non-symbiotic *Rhizobium* can utilize NO_3_^-^ and NH_4_^+^. We assume the same costs of NO_3_^-^ and NH_4_^+^ uptakes as those of legume’s, namely, $\text{13}.\text{5}\leq \overset{\text{N}}{\underset{R}{a}}(\text{1})\leq \text{17}$ and $\overset{\text{N}}{\underset{R}{a}}(\text{0})=\text{0}.\text{5}$. Non-symbiotic *Rhizobium* takes up various forms of C including glucose (C_6_H_12_O_6_) [29]. To take up glucose, *Rhizobium* uses ATP-binding cassette (ABC) transporters [30], which consume 2 ATP per glucose. For there are six C in a glucose, ATP cost of C for *Rhizobium* is then 0.33 mol ATP/mol C. We assume it as a baseline and let $\overset{\text{C}}{\underset{R}{a}}(\text{0})=\text{0}.\text{3}\text{3}$. With $\overset{\text{N}}{\underset{R}{a}}(\text{0})=\text{0}.\text{5}$, this leads to $r_{R}=\frac{\text{0}.\text{5}}{\text{0}.\text{3}\text{3}}=\text{1}.\text{5}\text{1}$. Also, our assumption $r_{R}=\frac{\overset{\text{N}}{\underset{R}{a}}(\text{1})}{\overset{\text{C}}{\underset{R}{a}}(\text{1})}$ implies, with $\text{13}.\text{5}\leq \overset{\text{N}}{\underset{R}{a}}(\text{1})\leq \text{17}$, that 8.94$\leq \overset{\text{C}}{\underset{R}{a}}(\text{1})\leq \text{1}\text{1}.\text{2}\text{5}$.

To sum up, if we use the median for $r_{L}(\text{1})$, we have


$$\begin{array}{c} r_{L}(\text{1})=\text{1}.\text{69}>r_{R}=\text{1}.\text{51},\;r_{R}=\text{1}.\text{51}>r_{L}(\text{0})=\text{0}.\text{0}\text{5}, \end{array}$$ (12)

i.e., the inequalities in Eq 6 are satisfied. It remains to verify the inequality in Eq 7, or, what amounts to the same thing, the inequality


$$\begin{array}{c} \overset{\text{N}}{\underset{R}{\overset{\text{N}}{\underset{R}{a}}(\text{1})\geq \hat{a}}}. \end{array}$$ (13)

As to the value of $\overset{\text{N}}{\underset{R}{\hat{a}}}$, namely, ATP cost of N-fixation, symbiotic *Rhizobium* fixes one molecule of aerial N_2_ to two molecules of ammonia (NH_3_) using nitrogenase. In this process, 16 ATP and 8 Fd_red_ are used [22]; here, it is thought that 2 ATP are required per Fd_red_ [31]. NH_3_ to NH_4_^+^ is zero cost. Thus, we assume that 32 mol ATP/ mol N_2_ is required, namely, $\overset{\text{N}}{\underset{R}{\hat{a}}}=\text{16}$. Comparing this figure to the possible range of $\overset{\text{N}}{\underset{R}{a}}(\text{1})$, $\text{13}.\text{5}\leq \overset{\text{N}}{\underset{R}{a}}(\text{1})\leq \text{17}$, we may conclude that Eq 13 is being satisfied. This result suggests that the condition for symbiosis as predicted by the comparative advantage argument is supported by the ATP cost data of host and symbiont.

### (b) Equilibrium values

Our model predicts various equilibrium values (Eqs 9–11). Here, we assess the values by checking two unit-free ratios: the supply ratio of C by legume $\frac{\overset{\text{C}*}{\underset{L}{y}}}{h}=\frac{r}{c_{L}+r}$ and that of N by *Rhizobium* $\frac{\overset{\text{N}*}{\underset{R}{y}}}{w^{*}}=\frac{c_{R}}{c_{R}+r}$, assuming that $c_{L}$ and $c_{R}$ are equal to the C:N ratios of legume and *Rhizobium*, respectively. Now, the C:N ratio of hairy vetch clover is 11:1, legume hay 17:1, and mature alfalfa 25:1 [32]; sweet clover 25.9:1 and blue lupin 20:1 [33]. We therefore assume that $c_{L}$ is between 11 and 26. The C:N ratio of soil microbes is 8:1 on average [32]. The C:N ratio of *Rhizobium meliloti*, however, is between 11:1 and 12:1 [34]. The $c_{R}$ value is thus likely between 11 and 12. It is our hypothesis that legume and *Rhizobium* trade under the exchange ratio *r* in the range of Eq 8. Thus, using our estimates $r_{R}=\text{1}.\text{5}\text{1}$ and $\text{1}.\text{5}\leq r_{L}(\text{1})\leq \text{1}.\text{8}\text{8}$, we assume *r* in the range 1.51≤r≤1.88. It is interesting to note that the theoretical cost of biological N fixation in units of C, namely $\frac{\overset{\text{N}}{\underset{R}{\hat{a}}}}{\overset{C}{\underset{L}{a}}}=\text{1}.\text{7}\text{7}$, falls within this range. Table 1 shows possible ranges of $\frac{\overset{\text{C}*}{\underset{L}{y}}}{h}$ and $\frac{\overset{\text{N}*}{\underset{R}{y}}}{w^{*}}$ for r=1.51 (the lower bound), r=1.69, and r=1.88 (the upper bound), assuming $\text{1}\text{1}\leq c_{L}\leq \text{2}\text{6}$ and $\text{1}\text{1}\leq c_{R}\leq \text{1}\text{2}$. As can be seen from the expressions, supply ratio of C (resp. N) increases (resp. decreases) as the exchange ratio *r* increases.

**Table 1:** Possible ranges of supply ratios of C and N.

	Meaning	Expression	Estimates	Reported values
$\frac{\overset{\text{C}*}{\underset{L}{y}}}{h}$	Supply ratio of C	$\frac{r}{c_{L}+r}$	0.05–0.12 (with r=1.51) 0.06–0.13 (with r=1.69) 0.06–0.14 (with r=1.88)	0.099–0.218 [35]
$\frac{\overset{\text{N}*}{\underset{R}{y}}}{w^{*}}$	Supply ratio of N	$\frac{c_{R}}{c_{R}+r}$	0.87–0.88 (with r=1.51) 0.86–0.87 (with r=1.69) 0.85–0.86 (with r=1.88)	0.93 [36]

Legume supplies in vegetative state about 9.9 to 21.8% of the C that it fixes to *Rhizobium* [35], whereas *Rhizobium* supplies over 93% of its fixed N to legume [36]. The predictions of the model are that legume supplies 5–14% of its fixed C and *Rhizobium* supplies 85–88% of its fixed N (see Table 1). Although the predicted ratio of C supply is slightly lower than the reported one, it seems that the tendency of a small supply ratio of C and a large supply ratio of N is reproduced here.

### (c) Effects of the C:N ratios of host and symbiont

By partially differentiating the supply ratios $\frac{\overset{\text{C}*}{\underset{L}{y}}}{h}$ by $c_{L}$ and $\frac{\overset{\text{N}*}{\underset{R}{y}}}{w^{*}}$ by $\;c_{R}$, we have:


$$\begin{array}{c} \frac{\partial }{{\partial c}_{L}}\frac{\overset{\text{C}*}{\underset{L}{y}}}{h}=\frac{\partial }{{\partial c}_{L}}\left(\frac{r}{c_{L}+r}\right)<\text{0}\text{ and }\frac{\partial }{\partial c_{R}}\frac{\overset{\text{N}*}{\underset{R}{y}}}{w^{*}}=\frac{\partial }{\partial c_{R}}\left(\frac{c_{R}}{c_{R}+r}\right)>\text{0}. \end{array}$$ (14)

Thus, the higher the value of $c_{L}$ is, the lower the supply ratio of C is; the lower the value of $c_{R}$ is, the lower the supply ratio of N is.

In general, $c_{L}$ increases with age [37]. For example, young alfalfa hay has a C:N ratio of 13:1, while that of mature alfalfa is 25:1 [32]. The C:N ratio of *R. meliloti* decreases with age [34], i.e., $c_{R}$ decreases with age. The two inequalities in Eq 14 then predict that the supply ratios of C and N both decrease with age (the change in the total amount of fixed N is indefinite because $\frac{\partial w^{*}}{\partial c_{L}}<\text{0}$ and $\frac{\partial w^{*}}{\partial c_{R}}<\text{0}$; see Table 2). The nodulation of legumes decreases with age [35], which may be explained by the predictions of decreasing trade with age.

**Table 2:** Sign of partial derivatives.

	Expression	$\frac{\partial }{\partial c_{L}}$	$\frac{\partial }{\partial c_{R}}$
$\frac{\overset{\text{C}*}{\underset{L}{y}}}{h}$	$\frac{r}{c_{L}+r}$	Negative	0
$\frac{\overset{\text{N}*}{\underset{R}{y}}}{w^{*}}$	$\frac{c_{R}}{c_{R}+r}$	0	Positive
$w^{*}$	$\frac{\left(c_{R}+r\right)h}{c_{R}(c_{L}+r)}$	Negative	Negative

It should also be noted that the C:N ratios of legumes are lower than those for other plants (for example, non-legume plants: barley straw 85:1, oat straw 70:1, corn stalks 60:1 [38]; legume plants: sweet clover 25.9:1 and blue lupin 20:1 [33]). The low $c_{L}$ values of legume suggest that legumes are more suitable for symbiosis with N-fixing partners than other plants.

## 4. Discussions

### (a) On the model

We have shown a comparative advantage microbial market model of C–N exchange in legume–*Rhizobium* symbiosis extending Schwartz and Hoeksema’s [9] model. A distinctive feature of our model is that it endogenously determines the ratio of fixed N to fixed C (i.e., the aspect-ratio of the Edgeworth box) and their supply ratios, given the body C:N ratios and some fixed C to N exchange ratio. ATP costs of uptake were used to determine the possible range of C to N exchange ratio. We have derived some implications of the model and assessed them against data accumulated over the last several decades. The following results were obtained: (i) The condition for the emergence of symbiosis predicted by the comparative advantage argument is supported by empirical ATP cost data of host and symbiont. (ii) The model predicts that supply ratio of C by legume is small and that of N by *Rhizobium* is large, which is consistent with reported figures. (iii) The model also predicts that the supply ratio of C decreases as the C:N ratio of legume increases; the supply ratio of N decreases as the C:N ratio of *Rhizobium* decreases, which explains the decrease in nodulation with aging. In our view, the result (ii) merits special attention because the characteristically imbalanced supply ratios are being explained by a few robust, stoichiometric quantities such as body C:N ratios and C to N exchange ratio. Of course, however, these predictions are still theoretical in nature, and further experimental validation is required. See also S2 Appendix for the supplementary discussion about the model.

### (b) On the physiology of exchange ratio (*r*) and ATP costs

We used ATP requirements as a common measure of biological constraint. The required numbers of ATP and ATP equivalents were counted using G3P, glucose, and NH_4_^+^ as the basis of metabolism The feasible range of the exchange ratio *r*, in particular, was determined by these ATP costs. Physiologically, no unified theory for this ratio *r* has yet been established [39], but it is generally considered to be governed by environmental carbon availability—particularly atmospheric concentrations [40]—as well as the abundance of other elements, such as phosphorus [41]. In this study, we treat this ratio as an exogenously given constant; otherwise, the equilibrium may not be well defined.

However, in addition to the processes described here, the period from the initiation to the breakdown of nodule formation includes other costly processes on the plant side, such as nodule development and maintenance. Based on the methodology we have developed, we aim to construct a more detailed model of the entire nodule life cycle that incorporates these processes. We also want to note that similar approaches may be possible for the study of other types of symbiosis, such as the relationship between plant and fungi.

### (c) Body mass adjustment process

In our static setting, we have derived the equilibrium of symbiosis *E* by the intersection of three rays. Here, we show a body mass adjustment process that converges to this equilibrium. Let $b_{R}(\sigma )$ be the ATP budget of *Rhizobium* of population size σ, and assume $\overset{\prime }{\underset{R}{b}}(\sigma )>\text{0}$ (the budget increases with the population size). The total supply of N by *Rhizobium* of population size σ is then $w(\sigma )=\frac{b_{R}(\sigma )}{\overset{N}{\underset{R}{a}}}$, and the total supply of C by legume is constantly $h=\frac{b_{L}}{\overset{C}{\underset{L}{a}}}$. The demand for C is the C-coordinate of the intersection of the trade line and the respective optimality line, so it is $\overset{C}{\underset{L}{x}}=\frac{c_{L}h}{c_{L}+r}$ for legume and $\overset{C}{\underset{R}{x}}(\sigma )=\frac{c_{R}rw(\sigma )}{c_{R}+r}\;$ for *Rhizobium*. Consider a process described by a first order ordinary differential equation


$$\begin{array}{c} \dot{\sigma }=F(\sigma ),\text{ where }F(\sigma )=h-\left(\overset{C}{\underset{L}{x}}+\overset{C}{\underset{R}{x}}(\sigma )\right)=h-\left(\frac{c_{L}h}{c_{L}+r}+\frac{c_{R}r}{c_{R}+r}w(\sigma )\right), \end{array}$$ (15)

i.e., the population size increases (resp. decreases) with the excess supply (resp. excess demand) of C. Since $\overset{\prime }{\underset{R}{b}}(\sigma )>\text{0}$, we have $w^{\prime }(\sigma )>\text{0}$, and $F^{\prime }(\sigma )<\text{0}$ for every σ, so this process is globally asymptotically stable. Also, we have $\dot{\sigma^{*}}=F\left(\sigma^{*}\right)=\text{0}$ if and only if $\sigma^{*}$ is such that $w\left(\sigma^{*}\right)=w^{*}=\frac{\left(c_{R}+r\right)h}{c_{R}\left(c_{L}+r\right)}$ (Eq 11), as expected.

## Supporting information

S1 Appendix Derivation of the equilibrium values (Eqs 9–11). (PDF)

S2 Appendix Supplementary discussion about the model. (PDF)

## References

[1] Cato MP. On Agriculture. In: Harrison F, editor. Complete Works of Cato the Elder. East Essex, UK: Delphi Publishing; 2016.

[2] Fred EB, Baldwin IL, McCoy E. The history of the *Leguminosae* in agriculture. Root Nodule Bacteria and Leguminous Plants. Madison, USA: University of Wisconsin Press; 1932. pp. 1–11.

[3] Mergaert P, Uchiumi T, Alunni B, Evanno G, Cheron A, Catrice O, et al. Eukaryotic control on bacterial cell cycle and differentiation in the *Rhizobium*-legume symbiosis. Proc Natl Acad Sci U S A. 2006;103(13):5230–5. doi: 10.1073/pnas.0600912103 16547129 PMC1458823

[4] Zgadzaj R, James EK, Kelly S, Kawaharada Y, de Jonge N, Jensen DB. A legume genetic framework controls infection of nodules by symbiotic and endophytic bacteria. PLoS Genetics. 2015;11(6):e1005280. doi: 10.1371/journal.pgen.1005280 PMC4456278 26042417

[5] Ramos J, Bisseling T. Symbiotic Nitrogen Fixation. In: Amâncio S, Stulen I. Nitrogen Acquisition and Assimilation in Higher Plants. Dordrecht, Holland: Kluwer Academic Publishers; 2004. pp. 99–131. doi: 10.1007/978-1-4020-2728-4_4

[6] Werner GDA, Strassmann JE, Ivens ABF, Engelmoer DJP, Verbruggen E, Queller DC, et al. Evolution of microbial markets. Proc Natl Acad Sci U S A. 2014;111(4):1237–44. doi: 10.1073/pnas.1315980111 24474743 PMC3910570

[7] Noë R, Hammerstein P. Biological markets: supply and demand determines the effect of partner choice in cooperation, mutualism and mating. Behav Ecol Sociobiol. 1994;35:1–11.

[8] Noë R, Hammerstein P. Biological markets. Trends Ecol Evol. 1995;10(8):336–9. doi: 10.1016/s0169-5347(00)89123-5 21237061

[9] Schwartz MW, Hoeksema JD. Specialization and resource trade: biological markets as a model of mutualisms. Ecology. 1998;79(3):1029–38. doi: 10.1890/0012-9658(1998)079[1029:sartbm]2.0.co;2

[10] Hoeksema JD, Schwartz MW. Expanding comparative-advantage biological market models: contingency of mutualism on partners’ resource requirements and acquisition trade-offs. Proc Biol Sci. 2003;270(1518):913–9. doi: 10.1098/rspb.2002.2312 12803905 PMC1691320

[11] Ricardo D. On the Principles of Political Economy and Taxation. London, UK: John Murray; 1817.

[12] Kummel M, Salant SW. The economics of mutualisms: optimal utilization of mycorrhizal mutualistic partners by plants. Ecology. 2006;87(4):892–902. doi: 10.1890/0012-9658(2006)87[892:teomou]2.0.co;2 16676533

[13] Cowden CC, Peterson CJ. A multi-mutualist simulation: applying biological market models to diverse mycorrhizal communities. Ecol Model. 2009;220:1522–33. doi: 10.1016/j.ecolmodel.2009.03.028

[14] Hammerstein P, Noë R. Biological trade and markets. Philos Trans R Soc Lond B Biol Sci. 2016;371(1687):20150101. doi: 10.1098/rstb.2015.0101 26729940 PMC4760201

[15] Streeter J, Wong PP. Inhibition of legume nodule formation and N2fixation by nitrate. Crit Rev Plant Sci. 1988;7(1):1–23. doi: 10.1080/07352688809382257

[16] Xia X, Ma C, Dong S, Xu Y, Gong Z. Effects of nitrogen concentrations on nodulation and nitrogenase activity in dual root systems of soybean plants. Soil Sci Plant Nutr. 2017;63(5):470–82. doi: 10.1080/00380768.2017.1370960

[17] von Liebig J. Die Chemie in irher Anwendung auf Agricultur und Physiologie. 7 ed. Braunschweig, Germany: Vieweg und Sohn; 1862.

[18] de Mazancourt C, Schwartz MW. A resource ratio theory of cooperation. Ecol Lett. 2010;13(3):349–59. doi: 10.1111/j.1461-0248.2009.01431.x 20455920

[19] Grman E, Robinson TMP, Klausmeier CA. Ecological specialization and trade affect the outcome of negotiations in mutualism. Am Nat. 2012;179(5):567–81. doi: 10.1086/665006 22504540

[20] Wakelin SA, Matson A, Wigley K, Waller L, Dickie IA, Whitehead D. High maintenance of rhizosphere soil C and N equilibrium regardless of plant species or species traits. Front. Soil Sci. 2021;1:762510. doi: 10.3389/fsoil.2021.762510

[21] Varian HR. Microeconomic Analysis. 3rd ed. New York: WW Norton Company; 1992.

[22] Buchanan BB, Gruissem W, Jones RL. Biochemistry and Molecular Biology of Plants. 2nd ed. Wiley; 2017.

[23] Schubert S. Nitrogen assimilation by legumes: processes and ecological limitations. Fert Res. 1995;42:99–107.

[24] Daniel RM, Smith IM, Phillip JAD, Ratcliffe HD, Drozd JW, Bull AT. Anaerobic growth and denitrification by *Rhizobium japonicum* and other Rhizobia. J Gen Microbiol. 1980;120:517–21.

[25] Fageria NK. The use of nutrients in crop plants. Boca Raton: CRC Press; 2009.

[26] Touraine B. Nitrate uptake by roots: transporters and root development. In: Amâncio S, Stulen I, editors. Nitrogen Acquisition and Assimilation in Higher Plants. Springer; 2004. pp. 1–34.

[27] Mantelin S, Touraine B. Plant growth-promoting bacteria and nitrate availability: impacts on root development and nitrate uptake. J Exp Bot. 2004;55(394):27–34. doi: 10.1093/jxb/erh010 14623902

[28] Britto DT, Kronzucker HJ. Futile cycling at the plasma membrane: a hallmark of low-affinity nutrient transport. Trends Plant Sci. 2006;11(11):529–34. doi: 10.1016/j.tplants.2006.09.011 17035071

[29] Stowers MD. Carbon metabolism in *Rhizobium* species. Annu Rev Microbiol. 1985;39:89–108. doi: 10.1146/annurev.mi.39.100185.000513 3904617

[30] Untiet V, Karunakaran R, Krämer M, Poole P, Priefer U, Prell J. ABC transport is inactivated by the PTS(Ntr) under potassium limitation in *Rhizobium leguminosarum* 3841. PLoS One. 2013;8(5):e64682. doi: 10.1371/journal.pone.0064682 23724079 PMC3665714

[31] Pate JS, Layzell DB. Energetics and biological costs of nitrogen assimilation. In: Stumpf PK, Conn EE, editors. The Biochemistry of Plants. San Diego, USA: Academic Press; 1990. pp. 1–42.

[32] USDA. 2011 Carbon to nitrogen ratios in cropping systems. USDA Natural Resources Conservation Service. https://www.nrcs.usda.gov/Internet/FSE_DOCUMENTS/nrcseprd331820.pdf

[33] Jensen HL. On the influence of the carbon:nitrogen ratios of organic material on the mineralization of nitrogen. J Agr Sci. 1929;19:71–82.

[34] Fred EB, Baldwin IL, McCoy E. Cultural and biochemical characteristics of the root nodule bacteria. Root Nodule Bacteria and Leguminous Plants. Madison, USA: University of Wisconsin Press; 1932. pp. 73–103.

[35] Atkins CA. Efficiencies and inefficiencies in the legume/*Rhizobium* symbiosis—a review. Plant Soil. 1984;82(3):273–84. doi: 10.1007/bf02184267

[36] Van Phi Hung N, Watanabe S, Ishikawa S, Ohtake N, Sueyoshi K, Sato T, et al. Quantitative analysis of the initial transport of fixed nitrogen in nodulated soybean plants using15N as a tracer. Soil Sci Plant Nutr. 2013;59(6):888–95. doi: 10.1080/00380768.2013.838742

[37] Wang Z, Lu J, Yang M, Yang H, Zhang Q. Stoichiometric Characteristics of Carbon, Nitrogen, and Phosphorus in Leaves of Differently Aged Lucerne (*Medicago sativa*) Stands. Front Plant Sci. 2015;6:1062. doi: 10.3389/fpls.2015.01062 26697029 PMC4673304

[38] USDA. 2014 Plant–microbe interaction—C:N ratio. Agronomy Tech Note 76. 2014. Available from: http://www.nrcs.usda.gov/wps/portal/nrcs/detail/nm/technical/?cid=nrcs144p2_068965

[39] Schwember AR, Schulze J, Del Pozo A, Cabeza RA. Regulation of symbiotic nitrogen fixation in legume root nodules. Plants (Basel). 2019;8(9):333. doi: 10.3390/plants8090333 31489914 PMC6784058

[40] Libault M. The carbon-nitrogen balance of the nodule and its regulation under elevated carbon dioxide concentration. Biomed Res Int. 2014;2014:507946. doi: 10.1155/2014/507946 24987690 PMC4058508

[41] Liu A, Contador CA, Fan K, Lam H-M. Interaction and regulation of carbon, nitrogen, and phosphorus metabolisms in root nodules of legumes. Front Plant Sci. 2018;9:1860. doi: 10.3389/fpls.2018.01860 30619423 PMC6305480

