# Supplementary material for: C–N exchange model of legume–Rhizobium symbiosis incorporating ATP budget constraints and energy–mass balance between the species
Source: PLoS One. 2026 May 22;21(5):e0349611. doi: 10.1371/journal.pone.0349611 (PMC13197078; doi:10.1371/journal.pone.0349611)
Supplement: S1 Appendix — (PDF) [file pone.0349611.s001.pdf]

## S1 Appendix. Derivation of the equilibrium values (Eqs. (9-11))

Let $h$ be the height of the box of Fig S1 (equivalent to Fig 4B of the main-text), which is the amount of C fixed by legume. Let $c_L$ and $c_R$ be the slopes of the optimality line of legume, $x_L^{\mathrm{C}} = \frac{k_L^{\mathrm{N}}}{k_L^{\mathrm{C}}} x_L^{\mathrm{N}}$, and that of *Rhizobium*, $x_R^{\mathrm{C}} = \frac{k_R^{\mathrm{N}}}{k_R^{\mathrm{C}}} x_R^{\mathrm{N}}$, respectively; i.e., $c_L = \frac{k_L^{\mathrm{N}}}{k_L^{\mathrm{C}}}$ and $c_R = \frac{k_R^{\mathrm{N}}}{k_R^{\mathrm{C}}}$. Now, $E$ is at the intersection of the optimality line $x_L^{\mathrm{C}} = c_L x_L^{\mathrm{N}}$ of legume and the trade line $x_L^{\mathrm{C}} = h - r x_L^{\mathrm{N}}$. By setting $c_L x_L^{\mathrm{N}} = h - r x_L^{\mathrm{N}}$, the legume's equilibrium demands are thus $x_L^{\mathrm{N}*} = \frac{h}{c_L + r}$ and $x_L^{\mathrm{C}*} = c_L x_L^{\mathrm{N}*} = \frac{c_L h}{c_L + r}$. The equilibrium supply of N by *Rhizobium* is $y_R^{\mathrm{N}*} = x_L^{\mathrm{N}*} = \frac{h}{c_L + r}$ and the equilibrium supply of C by the legume is $y_L^{\mathrm{C}*} = h - x_L^{\mathrm{C}*} = \frac{rh}{c_L + r}$. The equilibrium demands of *Rhizobium* are therefore $x_R^{\mathrm{C}*} = y_L^{\mathrm{C}*} = \frac{rh}{c_L + r}$ and $x_R^{\mathrm{N}*} = \frac{1}{c_R} x_R^{\mathrm{C}*} = \frac{rh}{c_R(c_L + r)}$. To summarize:

$$\left(x_L^{\mathrm{N}*}, x_L^{\mathrm{C}*}\right) = \left(\frac{h}{c_L + r}, \frac{c_L h}{c_L + r}\right),\ \left(x_R^{\mathrm{N}*}, x_R^{\mathrm{C}*}\right) = \left(\frac{rh}{c_R(c_L + r)}, \frac{rh}{c_L + r}\right) \tag{9}$$

and

$$y_L^{\mathrm{C}*} = x_R^{\mathrm{C}*} = \frac{rh}{c_L + r},\ y_R^{\mathrm{N}*} = x_L^{\mathrm{N}*} = \frac{h}{c_L + r}. \tag{10}$$

The equilibrium width $w^*$ of the box, which is the amount of N fixed by *Rhizobium*, is given by:

$$w^* = y_R^{\mathrm{N}*} + x_R^{\mathrm{N}*} = \frac{(c_R + r)h}{c_R(c_L + r)}. \tag{11}$$

The correspondence of these values to the elements of Edgeworth box is illustrated in Fig S1.

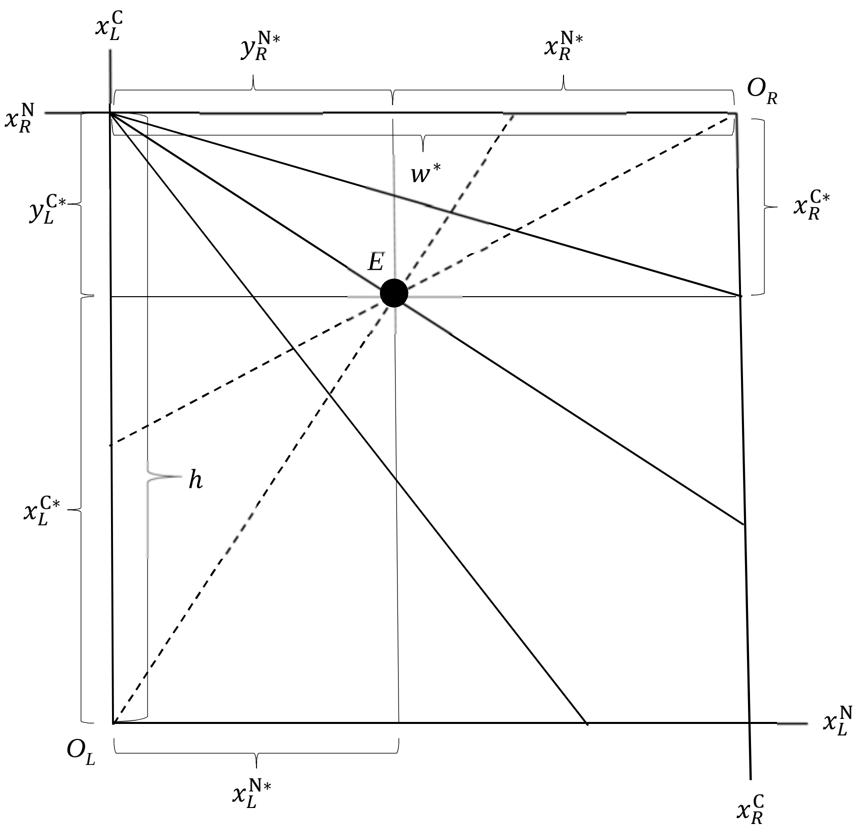


**Fig S1: Locations of various equilibrium values.**

The legume fixes an amount $h$ of C, from which it supplies $y_L^{C*}$ to *Rhizobium* and keeps $x_L^{C*}$. *Rhizobium* fixes an amount $w^*$ of N, from which it supplies $y_R^{N*}$ to legume and retains $x_R^{N*}$.
