# Supplementary material for: C–N exchange model of legume–Rhizobium symbiosis incorporating ATP budget constraints and energy–mass balance between the species
Source: PLoS One. 2026 May 22;21(5):e0349611. doi: 10.1371/journal.pone.0349611 (PMC13197078; doi:10.1371/journal.pone.0349611)
Supplement: S2 Appendix — (PDF) [file pone.0349611.s002.pdf]

S2 Appendix. Supplementary discussion about the model

Our model is based on the model of Schwartz and Hoeksema [1], with explicit specification of fitness function and linear constraint. The functional form of the fitness functions (Eqs 1, 3) is known as Leontief type in economics [2], and is also found implicitly [3] and explicitly [4] in biology, quoting Liebig's law of the minimum [5] as a rationale behind the theory. Grman et al [6] also use this form of fitness function with the elemental stoichiometry argument (this viewpoint already appeared in the "stoichiometric ray" or Kummel and Salant [7]). On the other hand, the linearity of budge lines (isolation lines) (Eqs 2, 4) comes from assumed constancy of ATP requirements in each species and situation. Therefore, although Schwartz and Hoeksema [1] discuss convex or concave variation of isolation lines, such a non-linearization may be difficult, if not impossible, for our empirical study.

The nature of exchange ratio r of C to N is also worth discussing. In our model, the exchange ratio r is assumed to be a uniform trading price of N. Wyatt et al [8] showed that such a uniform trading price ('linear proportional discrimination' [8]) is explained as an ESS (evolutionarily stable strategy) of their model. Grman et al [6] derived the trading price as a Nash bargaining solution [9], incorporating the similar approach of Akçay and Roughgarden [10] into the framework of Schwartz and Hoeksema [1]. Our treatment of ratio r is rather modest: we treat it as a given, exogenous variable that falls within a specific range. This enabled us to derive endogenous determination of the size of Edgeworth box, given the C:N ratios of host and symbiont.

That said, it is also interesting to note that, by treating this ratio, or a price of N, as being a variable parameter, and by differentiating the equilibrium value of the fixed N, w^*, with this price, we have:

$$\frac{\partial w^*}{\partial r} = \frac{h(c_L - c_R)}{c_R(c_L + r)^2} \gtreqless 0 \text{ if and only if } c_L \gtreqless c_R.$$

In reality, we have typically $c_L > c_R$ (recall our estimates $11 \leq c_L \leq 26$ and $11 \leq c_R \leq 12$). Therefore, *Rhizobium* will increase the supply of N if the price of N is raised, as suppliers of commodities do so in the ordinary markets. That this qualitatively rational feature results

from the empirical C:N ratios must be yet another support for the comparative advantage microbial market model.

References

1. Schwartz MW, Hoeksema JD. 1998 Specialization and resource trade: biological markets as a model of mutualisms. *Ecology* 79, 1029–1038.
2. Varian HR. 1992 *Microeconomic Analysis, 3rd ed.* New York, USA: WW Norton Company.
3. McGill B. 2005 A mechanistic model of a mutualism and its ecological and evolutionary dynamics. *Ecol Model* 187, 413–425.
4. de Mazancourt C, Schwartz MW. 2010 A resource ratio theory of cooperation. *Ecol. Let.* **13**, 349–359. (doi: 10.1111/j.1461-0248.2009.01431.x)
5. von Liebig J. 1862 *Die Chemie in irher Anwendung auf Agricultur und Physiologie, 7e ed* Braunschweig, Germany: Vieweg und Sohn.
6. Grman E, Robinson TMP, Klausmeier CA. 2012 Ecological specialization and trade affect the outcome of negotiation in mutualism. *Am. Nat.* 179, 567–581. (doi: 10.1086/665006)
7. Kummel M, Salant SW. 2006 The economics of mutualisms: optimal utilization of mycorrhizal mutualistic partners by plants. *Ecology* 87, 892–902. (doi.org/10.1890/0012-9658(2006)87[892:TEOMOU]2.0.CO;2)
8. Wyatt GAK, Kiers ET, Gardner A, West SAA. 2014 Biological market analysis of the plant–mycorrhizal symbiosis. *Evolution* 68, 2603–2618. (doi:10.1111/evo.12466)
9. Nash JF. 1950 The bargaining problem. *Econometrica* 18:155–162.
10. Akçay E, Roughgarden J. 2007 Negotiation of mutualism: Rhizobia and legumes. *P Roy Soc B-Biol Sci* 274:25–32.
